# Hippocampal Memory Recovery After Acute Stress: A Behavioral, Morphological and Molecular Study

**DOI:** 10.3389/fnmol.2018.00283

**Published:** 2018-08-17

**Authors:** Felipe Ignacio Aguayo, Macarena Tejos-Bravo, Gabriela Díaz-Véliz, Aníbal Pacheco, Gonzalo García-Rojo, Wladimir Corrales, Felipe Antonio Olave, Esteban Aliaga, José L. Ulloa, Ana M. Avalos, Luciano Román-Albasini, Paulina S. Rojas, Jenny Lucy Fiedler

**Affiliations:** ^1^Laboratory of Neuroplasticity and Neurogenetics, Department of Biochemistry and Molecular Biology, Faculty of Chemistry and Pharmaceutical Sciences, Universidad de Chile, Santiago, Chile; ^2^Laboratorio Farmacología del Comportamiento, ICBM, Facultad de Medicina, Universidad de Chile, Santiago, Chile; ^3^Department of Kinesiology, Faculty of Health Sciences, Universidad Católica del Maule, Talca, Chile; ^4^Facultad de Psicología, Universidad de Talca, Talca, Chile; ^5^Instituto de Ciencias Biomédicas, Facultad de Ciencias de la Salud, Universidad Autónoma de Chile, Santiago, Chile; ^6^Escuela de Química y Farmacia, Facultad de Medicina, Universidad Andres Bello, Santiago, Chile

**Keywords:** acute stress, learning and memory, hippocampus, dendritic spines, actin dynamics, RhoA/ROCK signaling pathway, LIMK, glutamate receptors

## Abstract

Several studies have shown that a single exposure to stress may improve or impair learning and memory processes, depending on the timing in which the stress event occurs with relation to the acquisition phase. However, to date there is no information about the molecular changes that occur at the synapse during the stress-induced memory modification and after a recovery period. In particular, there are no studies that have evaluated—at the same time—the temporality of stress and stress recovery period in hippocampal short-term memory and the effects on dendritic spine morphology, along with variations in *N*-methyl-D-aspartate (NMDA) and α-amino-3-hydroxy-5-methyl-4-isoxazolepropionic acid (AMPA) receptor subunits. The aim of our study was to take a multidimensional approach to investigate concomitant behavioral, morphological and molecular changes induced by a single restraint stress exposure (2.5 h) and a recovery period of 6 and 24 h in rats. We found that acute stress elicited a reduced preference to explore an object placed in a novel position (a hippocampal-dependent task). These changes were accompanied by increased activity of LIM kinase I (LIMK; an actin-remodeling protein) and increased levels of NR2A subunits of NMDA receptors. After 6 h of recovery from stress, rats showed similar preference to explore an object placed in a novel or familiar position, but density of immature spines increased in secondary CA1 apical dendrites, along with a transient rise in GluA2 AMPA receptor subunits. After 24 h of recovery from stress, the animals showed a preference to explore an object placed in a novel position, which was accompanied by a normalization of NMDA and AMPA receptor subunits to control values. Our data suggest that acute stress produces reversible molecular and behavioral changes 24 h after stress, allowing a full reestablishment of hippocampal-related memory. Further studies need to be conducted to deepen our understanding of these changes and their reciprocal interactions.Adaptive stress responses are a promising avenue to develop interventions aiming at restoring hippocampal function impaired by repetitive stress exposure.

## Introduction

Acute stress exposure may modify memory processes in several ways, depending on the extent, intensity and timing in which the stress event occurs (Joels et al., 2006). Stress effects are commonly mediated by the activation of the hypothalamic-pituitary-adrenal (HPA) axis and the release of glucocorticoids (Uchoa et al., 2014), which mediate fast actions in the brain that favor the release of glutamate from cortex and hippocampal areas (Moghaddam et al., 1994; Popoli et al., 2011) and strengthen synaptic transmission (Krugers et al., 2010). Although acute stress exerts beneficial effects on memory acquisition (Joels et al., 2006), it impairs memory retrieval (de Quervain et al., 1998). In paradigms that use object location memory, acute restraint stress and corticosterone (CORT) treatment before test trials has been shown to reduce exploration of novel objects and increase preference for familiar objects (Vargas-López et al., 2015). This evidence may indicate that stress and CORT impair the memory process. Converging with these evidences, another study found that restraint stress (1 h) elicited impaired memory acquisition, consolidation and retrieval in rodents, as assessed with object-recognition and object-location tasks (OLT; Li et al., 2012). These evidences suggest that acute stress may modulate hippocampus functioning by changing synaptic strength, thereby impacting cognitive process, including learning and memory (Middei et al., 2014). For instance, studies conducted in rodents have indicated that immediately after a single acute stress session—such as the exposure to the elevated platform (Cazakoff and Howland, 2010) or water tank (Kavushansky et al., 2006)—long-term potentiation (LTP) is impaired in the CA1 area, but enhanced in the dentate gyrus (DG) of the hippocampus (Kavushansky et al., 2006). Additionally, in rodents, a brief neck restraint stress facilitates LTP, but suppresses long-lasting depression (LTD) in the DG; effect that was related to the activation of both glucocorticoid and mineralocorticoid receptors (Spyrka et al., 2011). Although these data seem to be discordant, it is important to highlight that each type of stressor may influence particular brain circuits by modulating synaptic plasticity, which finally influences the animal’s behavior.

Some evidences have described that LTP and LTD induction can be produced by activation of *N*-methyl-D-aspartate (NMDA)-type glutamate receptors (Lüscher and Malenka, 2012). Moreover, α-amino-3-hydroxy-5-methyl-4-isoxazolepropionic acid (AMPA)-type glutamate receptors are redistributed in the early phases of LTP and LTD (Lüscher and Malenka, 2012). This process allows synapse potentiation or weakening, depending on whether the receptors are inserted or removed from the synaptic surface, respectively (Lüscher and Malenka, 2012). However, in mice, restraint stress during 1 h suppresses the induction of LTP and this was not related to a variation in the content of NMDA receptor subunits (NR1 and NR2B) or AMPA receptor subunits (GluA1 and GluA2) in a plasma membrane fraction obtained from hippocampus (Jin et al., 2015). To date there are no studies evaluating the effect of acute stress (i.e., a single exposure) on the levels of AMPA and NMDA receptors in synaptic fractions during the stress recovery period.

Furthermore, changes in LTP and LTD have been associated with increased spinogenesis and spine head growth (Engert and Bonhoeffer, 1999), whereas LTD has been associated with spine shrinkage and retraction (Zhou et al., 2004). Additionally, studies have reported a strong relationship between the number of mushroom-shaped spines in the hippocampus and memory formation (Mahmmoud et al., 2015). These morphological changes are related to signaling pathways that trigger the reorganization of actin filaments, which are the principal cytoskeletal constituent of spines (Bosch and Hayashi, 2012). However, there is not much information describing whether acute stress and its recovery period affect spine density in the hippocampus, in parallel with changes in the signaling pathways related to actin dynamics. *In vitro* studies have shown that spine growth involves the activity of Rho-GTPases (Luo, 2000; Nakayama et al., 2000). For instance, Rac1 activation stimulates actin polymerization and stabilizes dendritic spines by activating the downstream effectors p21-activated kinase (PAK), LIM kinase I (LIMKI) and inactivating cofilin, a potent actin-depolymerizing molecule (Nakayama et al., 2000; Calabrese et al., 2006). In contrast, RhoA-GTPase activity reduces the number of spines and their length (Nakayama et al., 2000; Nakayama and Luo, 2000) through activation of Rho serine/threonine kinase (ROCK), which regulates cytoskeleton dynamics by phosphorylating both the myosin light chain (MLC) and myosin phosphatase-targeting subunit 1 (MYPT1) of MLC phosphatase (Amano et al., 1996). Thus, changes in the activity of ROCK may define variations in myosin contraction of actin filaments and retraction of spines (Ryu et al., 2006).

Considering all these antecedents, our aim was to combine behavioral, morphological and molecular approaches to investigate the effect of a single acute stress exposure and recovery period on hippocampus-associated memory; changes that may be related to variations in dendritic spine density, along with variations in the activity of pathways related to actin dynamics and the levels of AMPA receptor and NMDA receptor subunits. This study may provide valuable insights about the neuroplastic mechanisms that occur in parallel with hippocampus-associated memory modifications after acute stress exposure.

## Materials and Methods

### Animals

Adult male Sprague-Dawley rats (320–350 g) were obtained from a stock maintained at the Faculty of Chemical and Pharmaceutical Sciences, Universidad de Chile. Animals were provided with food and water *ad libitum* and were maintained at 22°C with a controlled photoperiod of 12 h (lights on from 7:00 a.m. to 7:00 p.m.). Efforts were made to reduce both the number of animals used and their suffering. The rats were handled according to guidelines outlined and approved by the Ethical Committee of the Faculty of Chemical and Pharmaceutical Sciences (CBE2011-7-4), Universidad de Chile, and the Science and Technology National Commission (CONICYT), in compliance with the National Institutes of Health Guide for Care and Use of Laboratory Animals (NIH Publication, 8th Edition, 2011).

### Acute Stress Model

Rats were handled and stressed using published protocols, with minor modifications (Aguayo et al., 2018). Briefly, for morphological and biochemical analyses, rats were stressed by restraint in plexiglass tubes (25 × 8 cm) wide enough to allow comfortable breathing, but with restricted movement for 2.5 h (S 2.5; *n* = 10). In order to evaluate post-stress (PS) effects, another group of animals was stressed during 2.5 h and sacrificed 6 h (PS 6; *n* = 11) and 24 h after the stress period (PS 24; *n* = 13). At the end of the stress procedure, fecal output was determined. Unstressed animals were left undisturbed in their home cages in groups of 3–4 rats (C; *n* = 15). To evaluate fecal output in this group, the cage bedding was changed and after 2.5 h, the cage was inspected for fecal quantification. Hence, the estimation of fecal output corresponds to the mean value for a control group. For behavioral experiments, identical experimental groups were used. OLT (see below) was performed after the stress procedure (S 2.5; *n* = 8) and after 6 (PS 6; *n* = 8) or 24 h post stress (PS 24; *n* = 8). The unstressed group (C; *n* = 8) was submitted directly to the OLT.

### Object Location Task (OLT)

**Figure 1:**
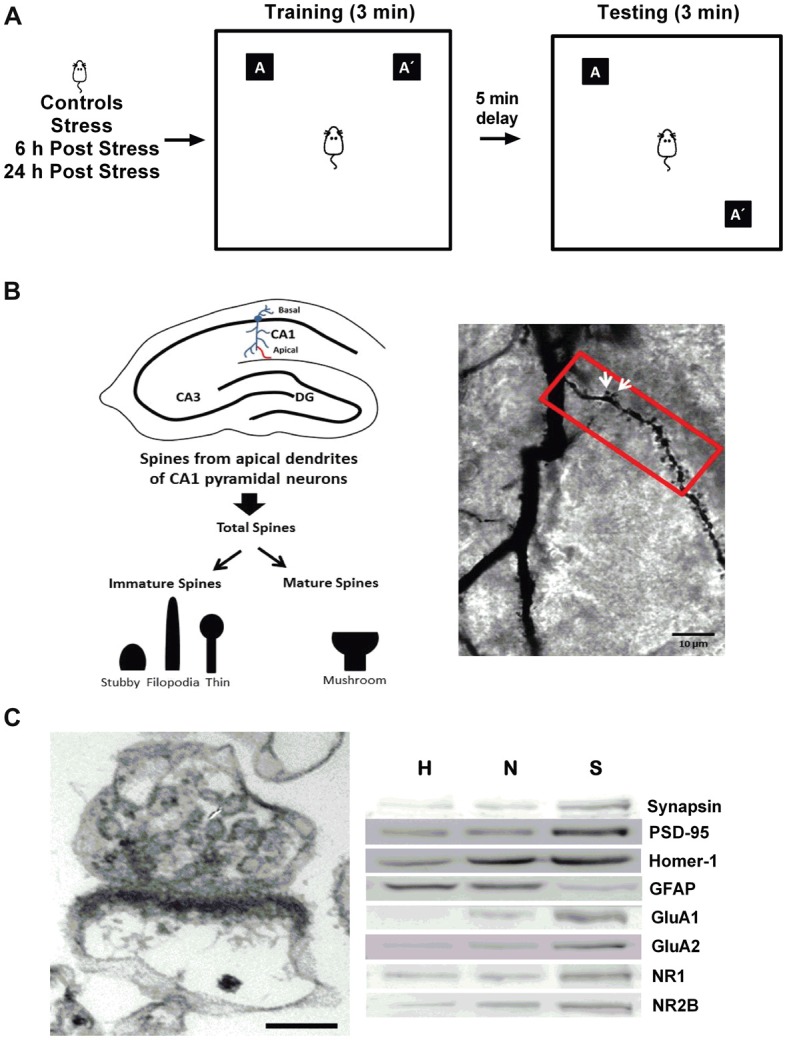
General methodology. **(A)** The object location task (OLT) was used as a readout of hippocampal-dependent memory. Rats were submitted to restraint stress, followed by 3 min of training phase. After a delay of 5 min, the location of one of the objects (A’) was changed, and rats were tested for exploration time. **(B)** Dendritic spines were analyzed in CA1 secondary apical dendrites across the first 80 mm segments and total spines were classified as mature (mushroom-shaped) or immature (thin-spines, filopodia-like and stubby-shaped), as indicated in the left panel. Golgi stain was performed in the CA1 region of rat hippocampi (right panel), and white arrows indicate mature spines.**(C)** Morphological and biochemical characterization of the synaptoneurosome-enriched fraction. Electron micrograph showing a representative synaptoneurosome, characterized by the presence of an axon terminal identified by synaptic vesicles associated with a resealed postsynaptic compartment, and a postsynaptic density; evidencing intact synapses. Scale bar: 0.1 μm (left panel). Western blotting analysis of the different fractions obtained during the preparation of the synaptoneurosome-enriched fraction (H, homogenate; N, nucleus; S, synaptoneurosome). The presynaptic protein synapsin, the postsynaptic proteins PSD-95 and Homer-1, the α-amino-3-hydroxy-5-methyl-4-isoxazolepropionic acid (AMPA receptor subunits (GluA1 and GluA2) and the *N*-methyl-D-aspartate (NMDA) receptor subunits (NR1 and NR2B) were enriched in the synaptoneurosome fraction. The marker of glia component, GFAP, was distributed mainly in H and N.

This test evaluates the ability of rodents to discriminate between a familiar and novel location of an object (Barker and Warburton, 2011). Rats were handled for a week and then habituated to the arena (60 × 60 cm square) in two daily sessions of 10 min for 2 days (Figure 1A). At the third day, animals were submitted to the training phase of the OLT. During this phase, two identical objects of 7 cm in diameter were located at adjacent corners of one side of the arena (locations A and A’, Figure 1A). Then, the animal was placed at the center of this arena and allowed to explore both objects for 3 min and the experimenter recorded the time spent in the exploration of each object. After a delay of 5 min, the testing phase began by placing one of the two objects in the original position (A) and the other one in a diagonal corner (A’). Considering that both objects in the test phase were equally familiar to the rat, the one in the new position (A’) was usually preferred by control rodents to explore. The rat was then placed in the center of the arena and the time that the rat explored each object was registered during 3 min. The position of the moved object was counterbalanced between rats. In the stressed group, rats were previously restrained for 2.5 h and then were allowed to move freely around their home cages during 30 min before the training, in order to avoid non-specific motor effects due to movement restriction (Vargas-López et al., 2015). In order to control the effect of stress on locomotor activity, we evaluated the total exploring time during both the training and the testing phases of the OLT. After the stress session, two groups of rats were allowed to rest in their home cages for either 6 or 24 h, and then submitted to the OLT. Data were expressed as a discrimination index (DI) determined with the formula: DI = (time spent in exploring the object in a novel location − time spent in exploring object in a familiar location)/(total exploration time)*100. The values range between −100 and 100; where a negative value indicates a preference for exploring the object in a familiar location and a positive value represents a preference for exploring the object in a novel location.

### Serum Corticosterone Levels

All the animals were sacrificed between 10:30 h and 13:00 h, with exception of the PS 6 h group, which was sacrificed between 17 h and 18 h. Trunk blood samples were collected for the determination of serum CORT levels. Hormone level determination was carried out using CORT ELISA Kit (Enzo, New York, USA; Cat. ADI-900-097), according to the kit’s instructions. CORT levels were not evaluated in animals that were assessed for the OLT.

### Golgi Staining and Evaluation of Dendritic Spine Density

Golgi staining was performed using the FD Rapid Golgi Stain kit (FD Neuro Technologies, Baltimore, MD, USA), following the manufacturer’s protocol. Analyses were performed according to standard protocols of our laboratory (Castañeda et al., 2015; Garcia-Rojo et al., 2017). Spines were counted in secondary apical dendrites of pyramidal neurons from the CA1 region, starting from the origin of the dendrite, along a distance of 80 μm (Figure 1B, left). A mushroom spine type was identified as such when its head diameter exceeded 0.6 μm and was classified as “mature” (white arrows); the remaining spines were classified as “immature,” and consisted mainly of stubby, filopodia-like and thin spines (Figure 1B).

### Preparation and Characterization of Hippocampal Homogenate and Synaptoneurosome-Enriched Fraction

The tissue was processed as we have previously described (Aguayo et al., 2018). Briefly, hippocampi were homogenized in 0.35 M sucrose, 10 mM HEPES pH 7.4, 0.25 mM dithiothreitol, protease inhibitor cocktail (Roche, Germany), 0.125 mM Na_3_VO_4_, 2 mM NaF and 0.25 mM sodium pyrophosphate. Samples were then centrifuged at 1,000× *g* for 10 min at 4°C and the obtained pellet corresponded to the nuclear fraction and the supernatant was considered as homogenate voided of nucleus. Part of this supernatant was sequentially filtered using decreasing pore sizes (100, 80, 30 and 10 μm; Millipore, Darmstadt, Germany). The final filtrate was centrifuged at 18,000× *g* for 20 min at 4° to obtain a pellet enriched in synaptoneurosomes (Figure 1C, left). Proteins in each fraction were determined using the bicinchoninic method (Pierce™ BCA Protein Assay Kit Thermo Fisher Scientific, MA, USA). All fractions were boiled immediately in sample loading buffer and stored at −80°C until western blotting analysis.

Aliquots of 30 μg of hippocampal homogenate and nuclear fraction or 15 μg of synaptoneurosomal enriched-fraction were mixed with Laemmli buffer, boiled and finally resolved on 10% SDS-polyacrylamide gels, and finally processed for western blot, according to the conditions described in Table 1. The analysis by western blotting of the synaptoneurosome (S) fraction revealed enrichment of the presynaptic protein synapsin and a reduced level in glia marker (GFAP). This fraction also showed an enrichment in postsynaptic markers such as PSD-95, Homer, GluA1, GluA2, NR1 and NR2B (Figure 1C).

**Table 1:** Primary antibodies and blocking conditions used.

Antigen	Description of Immunogen	Source, Host Species, Cat. #, RRID	Concentration used	Primary antibody and blocking solution
β-Actin	Syntetic actin N-terminal aa. DDDIAALVIDNGSGK	Sigma-Aldrich, mouse monoclonal, cat# A5316, RRID:AB_476743	0.1 ng/mL	3% non-fat milk—TBS 0.1% Tween-20
GFAP	Purified GFAP from pig spinal cord	Sigma-Aldrich, mouse, monoclonal, cat# G3893, RRID:AB_477010	1:1,000	1% non-fat milk—PBS 0.1% Tween-20
GluA1	Synthetic peptide corresponding to aa. 895–907 of rat GluA1 conjugated to hemocyanin	Synaptic Systems, mouse monoclonal, cat# 182 011, RRID:AB_2113443	1 ng/mL	1% BSA—PBS
GluA2	Recombinant protein of rat GluA2 (C terminus; aa. 836–883)	Synaptic Systems, rabbit polyclonal, cat# 182 103, RRID:AB_21113732	0.5 ng/mL	3% non-fat milk—PBS
Homer1	Recombinant protein of human homer (aa 1–186).	Synaptic Systems, rabbit polyclonal, cat# 160 003, RRID:AB_887730	1:1,000	5% BSA—TBS 0.1% Tween-20
LIMK	Synthetic Mouse LIMK1 C-terminal aa.627–647	Sigma-Aldrich, rabbit polyclonal, Cat# L2290, RRID:AB_260391	7 μg/mL	1% non-fat milk-TBS 0.1% Tween-20
pT508-LIMK	R-Y-P-T-V-V	Sigma-Aldrich, rabbit polyclonal, Cat# 4504460, RRID:AB_2491619	2 μg/mL	3% non-fat milk-TBS 0.1% Tween-20
MYPT1	Synthetic peptide corresponding to aminoterminal residues of human MYPT1	Cell Signaling Technology, rabbit polyclonal, Cat# 5143, RRID:AB_1642257	1:250	5% non-fat milk-TBS 0.1% Tween-20
pT853-MYPT1	Synthetic phosphopeptide corresponding to residues surrounding Thr853 of human MYPT1	Cell Signaling Technology, rabbit polyclonal, Cat# 4563S, RRID:AB_1031185	1:500	5% non-fat milk-TBS 0.1% Tween-20
NR1	Recombinant protein of NR1 (aa. 660–811)	Synaptic Systems, mouse monoclonal, cat# 114 011, RRID:AB_887750	1 ng/mL	3% non-fat milk—PBS
NR2A	Recombinant protein of mouse NR2A corresponding to aa. 1265–1464	Millipore, rabbit polyclonal, Cat# 07–632 RRID:AB_310837	2 ng/mL	1% BSA—PBS
NR2B	Synthetic peptide corresponding to aa. 42–60 of rat NR2B conjugated to hemocyanin	Synaptic Systems, rabbit polyclonal, cat# 244 103, RRID:AB_10805405	0.5 ng/mL	1% BSA—PBS
Synapsin	Synthetic peptide corresponding to AA 2–28 from rat Synapsin1 (UniProt Id: P09951)	Synaptic Systems, chicken polyclonal, cat# 106 006, RRID:AB_2622240	1:500	3% non-fat milk—PBS

### Statistical Analysis

Statistical analyses were performed using GraphPad Prism (GraphPad Software Inc., San Diego, CA, USA). Data are expressed as mean ± SEM and were processed for normality test distribution (D’Agostino-Pearson omnibus and Shapiro-Wilk test). Comparisons between two groups were analyzed with the Mann-Whitney U test and comparisons of three or more groups were analyzed with the Kruskal-Wallis test, followed by Dunn’s *post hoc* test.

## Results

### Acute Stress Increases Fecal Pellet Output and Serum Corticosterone Levels

**Figure 2:**
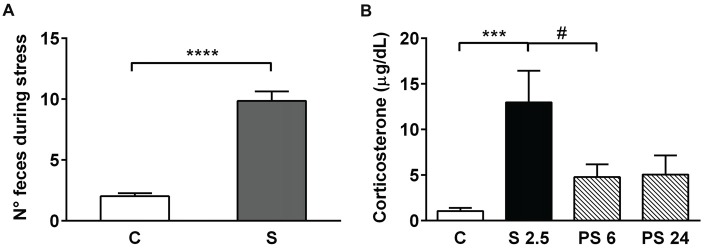
Acute restraint stress increases fecal pellet output and serum corticosterone (CORT) levels. **(A)** The graph represents mean ± SEM of the number of feces released by control unstressed rats (C, *n* = 15) and rats subjected to 2.5 h of restraint stress (S, *n* = 34). Data were analyzed by two-tailed Mann-Whitney test. **(B)** Acute restraint stress promoted an increase in serum CORT levels at the end of the stress session (S 2.5, *n* = 10) compared to the control group (C, *n* = 15), returning to basal levels at 6 and 24 h post-stress (PS 6 *n* = 11 and PS 24 *n* = 13, respectively). Data were analyzed by Kruskal-Wallis test followed by Dunn’s *post hoc* test. ***P < 0.001, ****P < 0.0001. Analysis between S 2.5 and PS 6 was conducted by two-tailed Mann-Whitney test, ^#^*P* < 0.05.

In order to evaluate the effectiveness of the stress protocol, we measured fecal pellet output in all the animals subjected to the 2.5 h stress procedure (Figure 2A) that was conducted in the morning. Mann-Whitney test analysis showed a significant increase in the number of fecal pellets voided during the 2.5 h of restraint, in comparison to unstressed animals (Figure 2A, S vs. C, *P* < 0.0001). We also evaluated CORT serum levels in samples obtained immediately after the stress session (S 2.5) and during the 6 h and 24 h stress recovery periods (PS 6 and PS 24). Kruskal-Wallis analysis demonstrated differences in experimental groups (*P* < 0.0001) and Dunn’s post-test showed that acute stress induced a five-fold increment in CORT levels in comparison to controls (S 2.5 vs. C, *P* = 0.0001; Figure 2B). The PS 6 group that was sacrificed during the afternoon (17-18 h) showed a significant reduction in CORT levels in comparison to the S 2.5 group (Mann-Whitney analysis), probably indicating that the afternoon surge in CORT secretion did not occur due to blunt stress-induced HPA-axis activity. Additionally, animals sacrificed 24 h after the acute stress exposure showed CORT levels similar to the control group (Figure 2B).

### Acute Stress-Induced Impairment of Object Location Memory Is Recovered 24 h After Stress

**Figure 3:**
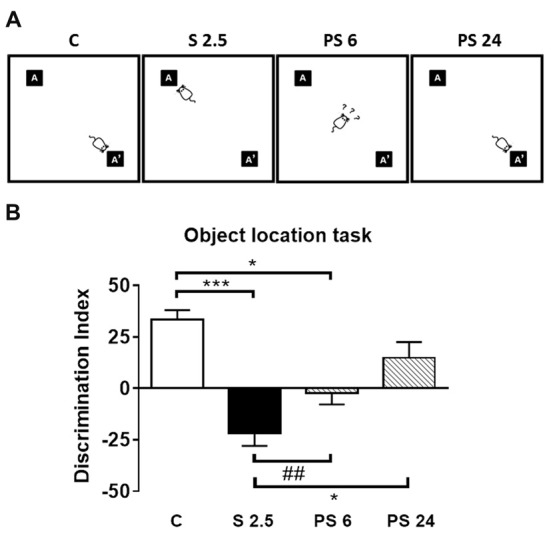
Acute stress does not influence locomotor activity, but triggers memory impairments in the Object Location Task (OLT). **(A)** Illustration of qualitative changes in OLT observed in the different experimental groups. **(B)** Discrimination index obtained in the OLT. The total exploration time that the rats spent in each object was obtained in control group (C, *n* = 8), immediately after restraint stress (S 2.5, *n* = 8) or during the recovery period (PS 6, *n* = 8 and PS 24, *n* = 8). The graph represents the effect of a single session of restraint stress on the performance in the OLT, measured as a discrimination index (DI). The stressed group of rats showed a negative DI compared to the control group. This condition was gradually reverted from PS 6 to PS 24 h during the recovery period. Data were analyzed by Kruskal-Wallis test followed by Dunn’s *post hoc* test. *P < 0.05, ***P < 0.001. Analysis between S 2.5 and PS 6 was conducted by two-tailed Mann-Whitney test, ^##^*P* < 0.01.

To evaluate the effects of a single restraint stress session on hippocampal functionality, we submitted rats to the OLT, which is a specific hippocampal-dependent task (Barker and Warburton, 2011) based on the natural tendency of animals to explore novel features in their environment (Vargas-López et al., 2015). This test evaluates the ability of rodents to discriminate between a familiar and novel location of an object (Figure 1A). Figure 3A illustrates qualitative changes in OLT detected in the different experimental groups and Figure 3B represents the DI expressed as percentage of time spent in a familiar position and new location over the total time spent in exploration. Kruskal-Wallis analysis showed differences between groups (*P* < 0.0001), and Dunn’s post-test indicated a decrease in the DI in stressed animals (C vs. S 2.5; *P* = 0.0001) and in the group evaluated at 6 h post stress (C vs. PS 6; *P* = 0.0163; Figures 3A,B). Moreover, 24 h after a single restraint stress, we observed that the DI increased with respect to the 2.5 h stress group (S 2.5 vs. PS 24; *P* = 0.0211), reaching a value that was not different from the control group (Figures 3A,B). Moreover, although we did not find differences between the S 2.5 and PS 6 groups with Dunn’s post-analysis, Mann-Whitney test revealed a significant difference between these groups (*P* = 0.0037). After 6 h of stress, the animals showed a DI value near zero, suggesting no preference for object location. In order to rule-out any locomotor alteration induced by experimental conditions, we determined the total exploration time. Kruskal-Wallis analysis showed no differences between the groups in total exploration time neither during the training phase nor during the testing phase of the OLT (Table 2). In whole, these data demonstrate that after a single restraint session, the performance of rats is impaired in the OLT and that spatial memory is partially recovered 24 h after stress.

**Table 2:** Total exploration time (s).

Experimental group	*N*	Training (Mean ± SD)	Testing (Mean ± SD)
C	8	31.5 ± 6.5	28.6 ± 9.7
S 2.5	8	29.1 ± 9.7	34.1 ± 8.7
PS 6	8	22.4 ± 5.7	24.6 ± 4.7
PS 24	8	27.5 ± 14.3	28.8 ± 7.3

### Acute Stress Triggers Transient Changes in Spine Density of Hippocampal CA1 Secondary Dendrites With No Changes in Levels of PSD-95 and Synaptophysin Synaptic Markers

**Figure 4:**
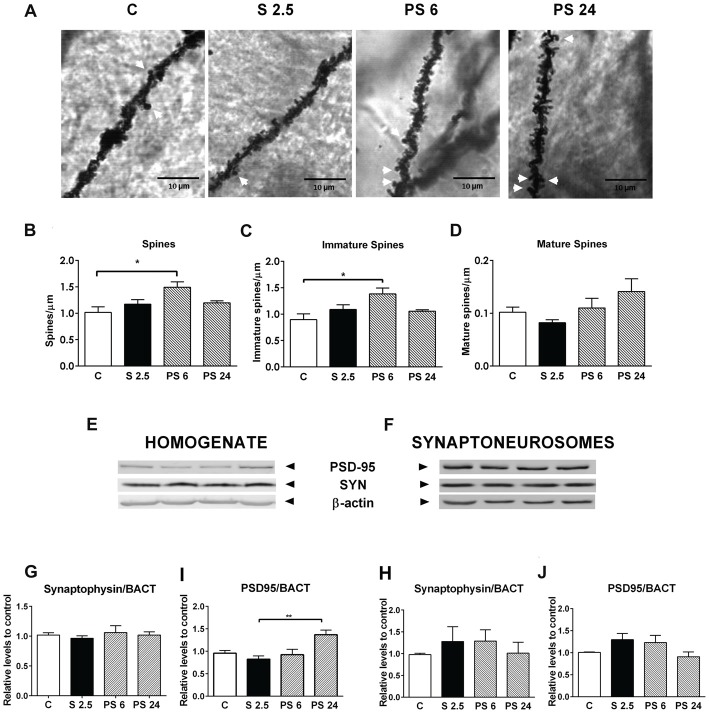
Acute stress triggers changes in dendritic spine density in CA1 neurons of hippocampus, which are not related to changes in the pre- and post-synaptic components. **(A)** Representative microphotographs of apical secondary dendrites of the CA1 region of hippocampus, corresponding to control group (C), stressed group (S 2.5) and post stress groups (PS 6 and PS 24). White arrows represent mushroom-shaped spines; scale bar represents 10 μm. **(B)** Total spine density, **(C)** Immature dendrite spine density and **(D)** Mature spine density in a dendritic segment of 80 μm in the control group (*n* = 7), S 2.5 (*n* = 5), PS 6 (*n* = 5) and PS 24 (*n* = 5) groups. **(E,F)** show a representative western blot of PSD-95 and synaptophysin (SYN) in both the homogenate and synaptoneurosome-enriched fractions. SYN levels in homogenate **(G)** and synaptoneurosomes **(H)** relative to β-actin. PSD95 levels in homogenate **(I)** and synaptoneurosomes **(J)** relative to β-actin. Data were analyzed by Kruskal-Wallis test followed by Dunn’s *post hoc* test. *P < 0.05, **P < 0.01.

To explore whether stress generates changes in dendritic spine density that may explain both the impairment and recovery of hippocampus functioning examined in the OLT, animals were subjected to the same stress procedure and sacrificed immediately after stress or the 6 or 24 h recovery period. Indeed, after 6 h of stress recovery, we found an increase in spine density along the dendritic segment, as shown in representative microphotographs of secondary dendrites of CA1 pyramidal neurons (Figure 4A, where mature spines are indicated by white arrows). For quantitative purposes, protrusions were classified as immature (stubby, filopodia and thin spines) or mature (mushroom-shaped spines), as indicated in Figure 1B. Kruskal-Wallis analysis indicated differences between groups (*P* = 0.0396) and Dunn’s *post hoc* analysis showed an increase in total spine density 6 h after stress (C vs. PS 6, *P* = 0.0264), change that was recovered to control levels 24 h after the stress session (Figure 4B). Interestingly, immature spine density also increased 6 h after stress (Figure 4C; Kruskal Wallis *P* = 0.0416, C vs. PS 6, *P* = 0.026) and also recovered to control levels 24 h after stress. On the other hand, mature spine density was not affected either by stress or during the two recovery periods (Figure 4D). Next, we decided to correlate these morphological changes with variations in the levels of pre- and post-synaptic markers in both the hippocampal homogenate and the synaptoneurosome-enriched fraction. Representative immunowestern blots of these fractions are shown in Figures 4E,F). The pre-synaptic marker synaptophysin (SYN) did not show variation either in hippocampal homogenate (Figure 4G) or synaptoneurosome fraction (Figure 4H). On the other hand, the levels of the post-synaptic marker PSD-95 changed with treatments (Kruskall-Wallis *P* = 0.009) showing an increase 24 h after stress in hippocampal homogenate (Dunn’s *P* < 0.0099, Figure 4I), but constant levels in the synaptoneurosome fraction in all experimental conditions (Figure 4J).

### Acute Stress Favors Signaling Associated to Actin Remodeling in Synaptoneurosomes From Hippocampus

**Figure 5:**
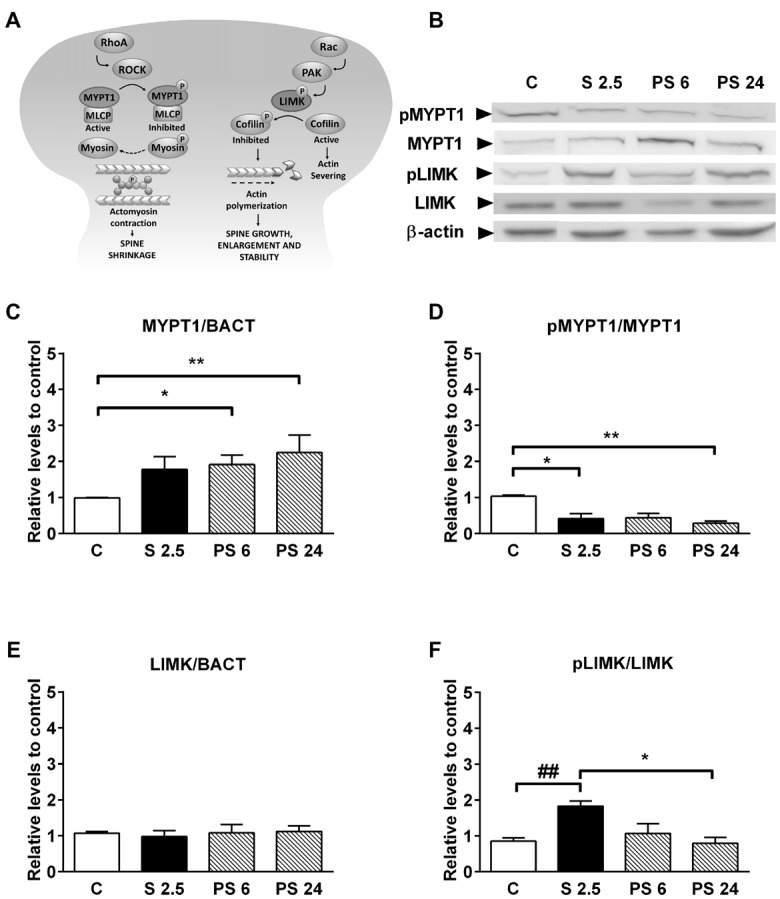
Acute stress promotes the activation of pathways that promote actin polymerization. **(A)** Diagram showing that RhoA and Rac small GTPases have opposite effects on dendritic spine stability. RhoA activates Rho-associated protein kinase (ROCK), which in turn phosphorylates a large number of downstream effectors that affect cytoskeleton dynamics. ROCK can inactivate the myosin phosphatase-targeting subunit 1 (MYPT1) of the myosin light chain (MLC) phosphatase and, indirectly, may increase the MLC phosphorylation state (Amano et al., 1996). This may favor actomyosin contractility, causing spine collapse and synapse loss. On the other hand, Rac activates LIM kinase (LIMK), which phosphorylates and inhibits cofilin—a potent actin-depolymerizing molecule—and therefore, alters spine actin turnover. **(B)** Representative immunoblots for pMYPT1 (T 853), MYPT1, pLIMK (T 508) and LIMK in the synaptoneurosome fraction. β-actin was used as a loading control. **(C)** Graph represents MYPT1 levels and **(D)** the pMYPT1/MYPT1 ratio. Controls (C, *n* = 7); animals stressed during 2.5 h that were sacrificed immediately (S 2.5, *n* = 5); or sacrificed 6 (PS 6, *n* = 6) and 24 h after stress (PS 24, *n* = 7). **(E)** Graph represents LIMK levels and **(F)** the pLIMK/LIMK ratio. Controls (C, *n* = 7); animals stressed during 2.5 h that were sacrificed immediately (S 2.5, *n* = 5); or sacrificed 6 (PS 6, *n* = 6) and 24 h post-stress (PS 24, *n* = 6). Data were analyzed by Kruskal-Wallis test followed by Dunn’s *post hoc* test. *P < 0.05, **P < 0.01. Analysis between C vs. S 2.5 was conducted by two-tailed Mann-Whitney test, ^##^*P* < 0.01.

Considering that the dynamics of actin cytoskeleton in dendritic spines is associated with the RhoA-ROCK signaling pathway (Figure 5A, diagram), we evaluated changes in the levels of MYPT1 and its phosphorylated form in synaptoneurosome fractions from hippocampus as a readout of ROCK activity. Representative immunoblots are shown in Figure 5B. Kruskal-Wallis analysis showed differences in total levels of MYPT1 relative to β-actin (*P* = 0.0075), and Dunn’s post-test revealed an increase of approximately 90% over control levels 6 h after stress (C vs. PS 6, *P* = 0.016, Figures 5B,C); variation that was sustained 24 h after stress (C vs. PS 24, *P* = 0.0064, Figure 5C). The activation of the RhoA–ROCK pathway was assessed by changes in Thr853 phosphorylation of MYPT1 (Figure 5A, diagram). We detected that treatments triggered variations in the pMYPT1/MYPT1 ratio (Kruskal-Wallis analysis *P* = 0.0037) and Dunn’s analysis indicated that this ratio decreased by approximately 60% relative to controls after 2.5 h of stress (C vs. S 2.5, *P* = 0.0147), reduction that was maintained until 24 after stress (C vs. PS 24, *P* = 0.002, Figure 5D). These data indicate that stress triggers a reduction in ROCK activity. We also evaluated the levels of LIMK1 and phosphorylated LIMK1 (Thr508), which is considered an *in vitro* effector of Rac GTPases (Edwards et al., 1999; Figure 5A, diagram). Although LIMK levels relative to β-actin were not affected by treatments (Figure 5E), we found that the pLIMK/LIMK ratio in the stress group was higher compared to PS 24 (Kruskal-Wallis *P* = 0.0435, Dunn’s post-test S 2.5 vs. PS 24, *P* < 0.0415; Figure 5F). Moreover, although we did not find differences between control and S 2.5 groups with Dunn’s post-analysis, Mann-Whitney test revealed a significant difference between these groups (*P* = 0.003). These results demonstrate that acute stress reduces ROCK activity and therefore, reduces the phosphorylated form of the MLC phosphatase-regulating subunit (Figure 5A, diagram) during both the stress and recovery periods. In contrast, LIMK activity increases transiently at synapses during the stress session. These modifications may favor actin polymerization by inhibiting cofilin, thereby promoting the generation filopodia-like spine precursors and favoring spine enlargement, growth and stability (Figure 5A, diagram).

### Acute Stress Induces Differential Expression of NMDA and AMPA Receptor Subunits in the Hippocampus

Our results have demonstrated that the acute stress-induced impairment of hippocampal function is recovered 24 h after stress; modifications that were accompanied by variations in signaling that promote actin dynamics by favoring actin polymerization. In order to complement these observations, which may explain both the deficit in hippocampal function and its recovery, we also examined whether a single stress session modified the levels of NMDA and AMPA receptor subunits in both hippocampal homogenate and synaptoneurosome fraction.

**Figure 6:**
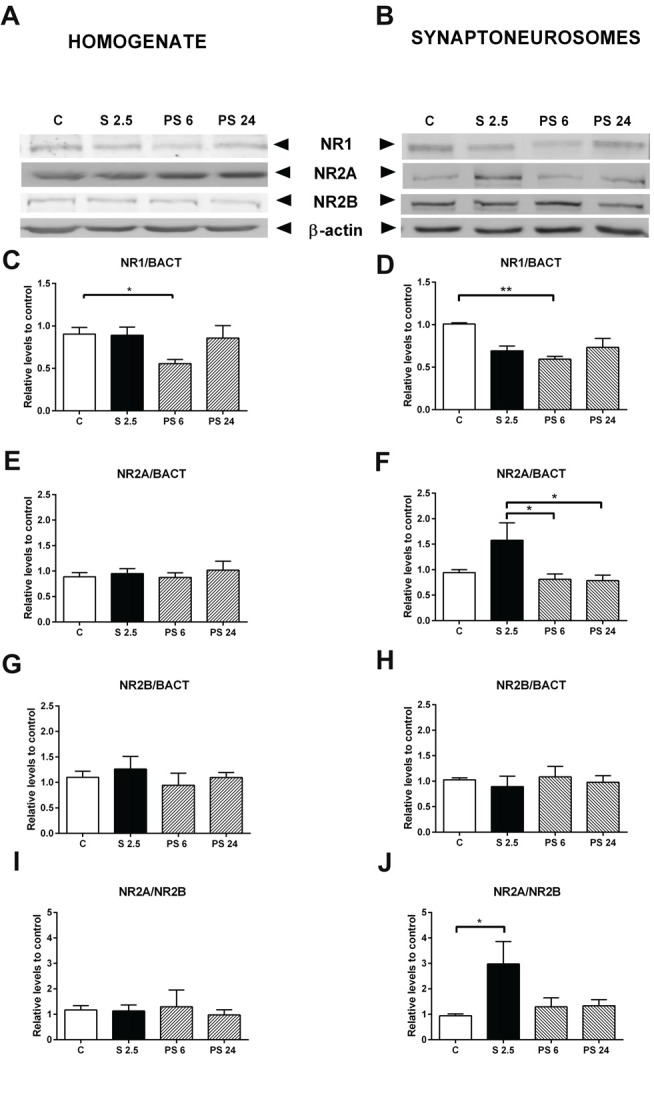
Effect of acute stress on NR1, NR2A and NR2B receptor subunit levels in total homogenate and synaptoneurosome fractions. Representative immunoblots for NR1, NR2A and NR2B in the homogenate **(A)** and synaptoneurosome fractions **(B)**. β-actin was used as a loading control. Graphs represent NR1 levels in homogenate **(C)** and synaptoneurosomes **(D)**, NR2A levels in homogenate **(E)** and synaptoneurosomes **(F)** and NR2B levels in homogenate **(G)** and synaptoneurosomes **(H)**. NR2A/NR2B ratio in homogenate **(I)** and synaptoneurosomes **(J)**. Control (C, *n* = 8); animals stressed during 2.5 h that were sacrificed immediately (S 2.5, *n* = 5); or sacrificed 6 (PS 6, *n* = 5) and 24 h post-stress (PS 24, *n* = 7). Data were analyzed by non-parametric Kruskal-Wallis test followed by Dunn’s *post hoc* test. *P < 0.05, **P < 0.01.

Representative immunowestern blots indicate changes in the levels of NMDA receptor subunits (Figures 6A,B). Kruskal-Wallis analysis indicated that treatments affected NR1 levels in homogenates (*P* = 0.046), and Dunn’s post-test revealed a significant reduction of almost 40% in the PS 6 group (C vs. PS 6, *P* = 0.043, Figure 6C), returning to basal levels 24 h after stress. A similar pattern was also detected in the synaptoneurosome fraction, i.e., a 40% reduction in NR1 levels 6 h after stress (Kruskal-Wallis *P* = 0.0095; C vs. PS 6, *P* = 0.0059), returning to basal levels 24 h after stress (Figure 6D). Although NR2A levels in hippocampal homogenate did not change (Figure 6E), we detected effect of treatments on NR2A levels in synaptoneurosomes (Kruskal-Wallis *P* = 0.0161). Dunn’s post-test revealed that NR2A levels increased by approximately 60% at the end of the stress period, compared with the 6 h and 24 h recovery periods (S 2.5 vs. PS 6, *P* = 0.0193; S 2.5 vs. PS 24, *P* = 0.0353, Figure 6F). In contrast, no significant variations were observed in NR2B subunit levels in both homogenate and synaptoneurosome fractions (Figures 6G,H). Consistently, the NR2A/NR2B ratio did not vary in the homogenate (Figure 6I), but increased with the stress session in synaptoneurosomes (Kruskal-Wallis, *P* = 0.0362, Dunn’s C vs. S 2.5, *P* = 0.0118, Figure 6J).

**Figure 7:**
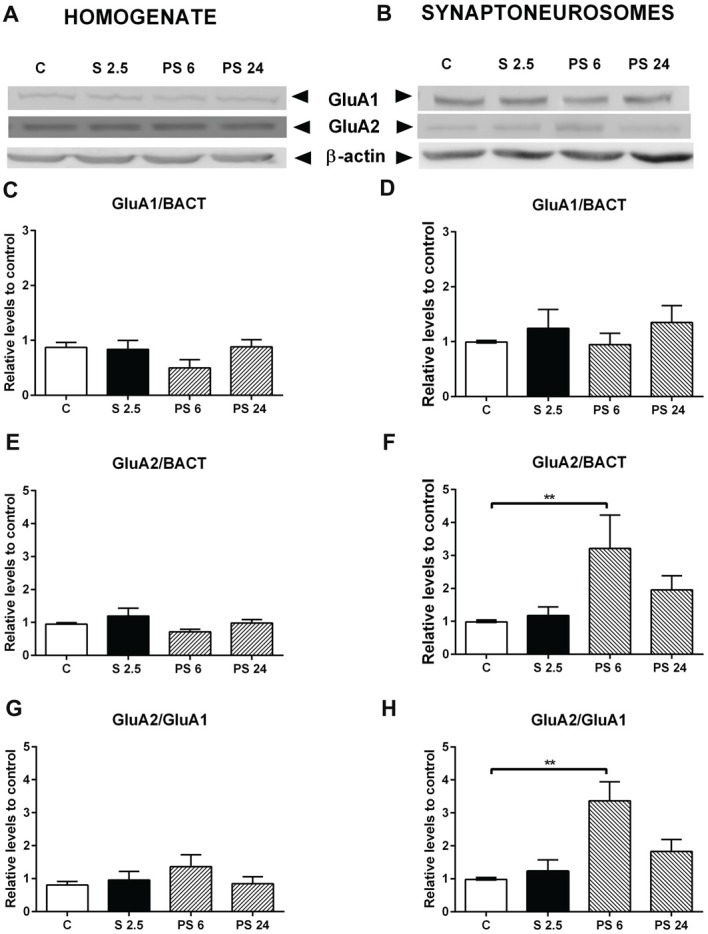
Effect of acute stress on GluA1 and GluA2 receptor subunits in total homogenate and synaptoneurosome fractions. Representative immunoblots for GluA1 and GluA2 in the homogenate **(A)** and synaptoneurosome fractions **(B)**. β-actin was used as a loading control. Graphs represent the levels of GluA1 in homogenate **(C)** and synaptoneurosomes **(D)**. Control (C, *n* = 6); animals stressed during 2.5 h that were sacrificed immediately (S 2.5, *n* = 4); or sacrificed 6 (PS 6, *n* = 4) and 24 h post-stress (PS 24, *n* = 6). GluA2 levels in homogenate **(E)** and synaptoneurosomes **(F)**. GluA2/GluA1 ratio in homogenate **(G)** and synaptoneurosomes **(H)**. Data were analyzed by non-parametric Kruskal-Wallis test followed by Dunn’s *post hoc* test. Control (C, *n* = 6); animals stressed during 2.5 h that were sacrificed immediately (S 2.5, *n* = 4); or sacrificed 6 (PS 6, *n* = 4) and 24 h post-stress (PS 24, *n* = 6). Data were analyzed by non-parametric Kruskal-Wallis test followed by Dunn’s *post hoc* test. **P < 0.01.

A similar analysis was conducted for AMPA receptor subunits and a representative immunowestern blot of GluA1 and GluA2 in homogenate and synaptoneurosomes are shown in Figures 7A,B, respectively. Quantitative analysis showed that GluA1 subunit levels in homogenate and synaptoneurosome were insensitive to the stress and the recovery period (Figures 7C,D). Although we did not observe variations in GluA2 levels in the homogenate (Figure 7E), we found a rise after 6 h of stress in the synaptoneurosome fraction (Kruskal-Wallis *P* = 0.0191, Dunn’s C vs. PS 6, *P* = 0.0009, Figure 7F); a variation that returned to control levels in the PS 24 group. We also observed that the GluA2/GluA1 ratio did not change in the homogenate (Figure 7G); nonetheless this ratio increased almost two-fold 6 h after stress in synaptoneurosomes, compared to the control group (Kruskal-Wallis *P* = 0.0095; Dunn’s C vs. PS 6, *P* = 0.0046) and returned to basal levels 24 h after stress (Figure 7H).

## Discussion

Stress is an adaptive response to different stimuli that comprises multiple interacting mediators that influence the structural and functional plasticity of the brain, processes known as neuroplasticity (McEwen and Gianaros, 2010). Under extreme conditions, the stress response is insufficient, favoring a maladaptive response that predisposes an individual to develop numerous pathologies, such as anxiety and depressive disorder (Otte et al., 2016). The study of how stress affects the hippocampus is particularly relevant considering that this structure is not only involved in memory, but also in the negative regulation of the HPA axis activity under stress (Kim et al., 2015). Here, we provided evidences that a single stress session produces a response with a particular kinetics that affects behavior, morphology and synaptic components of rat hippocampus. Immediately after stress, we observed a strong reduction in hippocampal short-term memory, change that was coincident with a rise in actin-dynamics (rise in pLIMK and reduction in ROCK activity) and NR2A subunit levels of NMDA receptors, but not with an evident change in spine morphology of CA1 neurons. However, 6 h after stress exposure, animals showed—similarly to the training phase in the OLT—no discrimination for an object located at a familiar or novel position; phenomena which may indicate some level of recovery in comparison to stressed animals. This variation was coincident with a rise in immature forms of dendritic spines, accompanied by a rise in the GluA2/GluA1 ratio in the synaptoneurosome fraction, but not in the hippocampal homogenate, and suggests an increment of AMPA receptors at the synapse. Surprisingly, 24 h after stress, short-term memory was recovered, the morphological changes were reversed, and NMDA and AMPA receptor subunit levels returned to the control condition. The knowledge of the mechanisms that trigger adaptive recovery of hippocampal short-term memory, the structural morphology of neurons and levels of glutamate subunit receptors may be useful to revert the consequences of maladaptive responses involved in mood stress-related disorders.

### Acute Stress Exposure Produces Impairment of Hippocampal Short-Term Memory, Which Occurs in Parallel With a Rise in NR2A Subunits in Synaptoneurosomes

The extent, timing and intensity of exposure to a stressor are important factors that modulate learning and memory (Joels et al., 2006). In contrast to chronic stress, which consistently impairs learning and memory processes, acute stress may exert positive or negative actions on memory formation (i.e., memory encoding and consolidation) and memory retrieval (Joels et al., 2006). Studies have reported that acute stress differentially affects diverse forms of hippocampal-dependent memory, impairing spatial memory and object recognition memory, but enhancing other types of hippocampus-based memory (Barker et al., 2007; Barker and Warburton, 2011; Kim et al., 2015). Studies that evaluated spatial learning and memory have used food-deprived rodents that are then exposed to a radial arm maze, in which the animal has the chance to locate food pellets in the arms (food reward). After recovering for 24 h from a brief intermittent tail shock, animals trained in this radial maze task display a preference to consume food pellets in the familiar arm (their first arm entry) instead of those located in a novel position (Shors, 2004). On the other hand, another report indicated that acute audiogenic stress lasting 30–60 min impairs the retention of spatial memory before training in the Morris water maze (Kim et al., 2007). In addition, stress seems to differentially impact specific phases of hippocampal memory. For instance, one report has indicated that after acute inescapable restraint-tail shock stress, rats markedly exhibit impaired recognition memory when a delay of 3 h occurred between the familiarization and test phases, but not when the delay was of 5 min (Baker and Kim, 2002). According to these results, the authors indicated that stress causes memory retrieval impairment (Baker and Kim, 2002), but other studies have posed the idea that stress induces transient novelty avoidance, rather than retrieval impairments (Vargas-López et al., 2015).

In the present study, we used the OLT and determined that a single acute stress exposure produces impairment in object location memory associated with the preference for an object in a novel place. Even if these data may also be interpreted as novelty avoidance, we are inclined for memory impairment because several studies have reported that stress alters LTP induction in the hippocampus, a form of synaptic plasticity that probably underlies the neural mechanism of learning and memory storage information (Whitlock et al., 2006). Interestingly, some *in vitro* experiments have evidenced that LTP is impaired in the CA1 area when the tissue is obtained immediately after acute inescapable restraint-tail shock stress (Kim et al., 1996). Another study reported an enhancement of LTP, but a suppression of LTD, in hippocampal slices obtained from animals under a brief neck restraint stress (10 min); importantly, this study was carried out after 3.5 h of stress (Spyrka et al., 2011). These evidences suggest that the opposing effect of stress on LTP may be related to the effect of the time elapsed between the stress and the electrophysiological recording.

A number of lines of evidence have suggested that the presence of NR2B subunits favors LTP induction, while the overexpression of NR2A subunits reduces the induction of LTP in neuronal cultures (Barria and Malinow, 2005). Additionally, when the NR2A/NR2B ratio is higher, a stronger stimulation is required to induce LTP, reviewed in Yashiro and Philpot (2008). Existing data suggest that overexpression of NR2A subunits specifically abolishes 3–5 Hz frequency-induced LTD in CA3-CA1 synapses, without affecting 100 Hz LTP or 1 Hz LTD; these changes were proposed to be essential for consolidating long-term memory traces in the brain (Cui et al., 2013). The relationship between glutamate receptor and memory processes has also been evaluated. For instance, mice overexpressing NR2A exhibit impairment in long-term memory, but not in short-term memory tests, suggesting that the consolidation process is compromised (Cui et al., 2013). Consequently, the deficit in consolidation has been detected in several different tasks, such as novel object recognition, contextual fear conditioning, cued fear conditioning and spatial plus-water maze; but the OLT was not evaluated in this study (Cui et al., 2013). Considering these facts, it is plausible that stress-induced impairment in OLT may be associated with a rise in the NR2A/NR2B subunit ratio of NMDA receptors in the synaptoneurosome fraction, and that during stress, NMDA receptors containing NR2A are delivered at synaptic sites. In line with this idea, hippocampal slices from adult rats briefly incubated with CORT were shown to trigger facilitation of LTP and LTD and this effect disappeared 1 h after the hormone was removed (Tse et al., 2011). Interestingly, the suppression of LTP facilitation was associated with a rise in the exposure of NR2A-containing receptors in the membrane (Tse et al., 2011). A recent study in hippocampal neuronal cultures exposed to CORT showed that the distribution and dynamics of NMDA receptor subtypes at the plasma membrane changed and that CORT produced a delayed and positive effect in the content of GluA1 AMPA receptors at the synapse (Mikasova et al., 2017). This study was conducted with a timing after CORT addition that is not comparable to our stress model (Mikasova et al., 2017).

In our study, the detrimental effect of acute stress in the OLT occured in parallel with the rise in NR2A subunit levels; this variation that has been reported to reduce LTP (Foster et al., 2010) and in our case, may explain the short-term impairment in memory. Moreover, these modifications were not accompanied by changes in dendritic spine density or morphology. Nonetheless, we detected changes that promote F-actin formation (rise in the pLIMK/LIMK ratio) and probably, also weaken acto-myosin interaction (reduction of ROCK activity), thereby favoring spine growth and stability.

### Dendritic Spine Density and GluA2 Levels Increase During the Stress Recovery Period and Occur in Parallel With a Recovery of Object Location Preference

The number of spines along a dendrite is considered to reflect connectivity among neurons through glutamate neurotransmission. Evidence has indicated that small, thin spines are more motile and form weak synapses; while mushroom spines are more stable, and are associated with strong synapses that have prominent postsynaptic densities and AMPA receptor density (Kasai et al., 2003). Dendritic spines are highly dynamic structures and provide a substrate to be reshaped under stimulus. Only a few studies have investigated the influence of acute stress on dendritic morphology in the hippocampus. Our study evidences for the first time, that the abundance of dendritic spines changes during stress and the recovery period by showing that spine density—mainly of immature forms—rises 6 h after a single stress session. As mentioned before, the change in spine morphology and number is mainly dependent on variations in globular and filamentous actin (Lei et al., 2016). Interestingly, in the present study we detected a rise in spine density that was preceded by a rise of LIMK activity and reduction of ROCK activity; kinases that are involved in actin dynamic remodeling (Nakayama et al., 2000; Nakayama and Luo, 2000). LIMK-1 is an ADF/cofilin specific kinase enriched in dendritic spines and LIMK-1^(−/−)^ neurons display thin spines, with a reduction in head volume, resulting in postsynaptic densities with decreased area (Meng et al., 2002). Thus, it is plausible that the higher activity of LIMK detected during the stress exposure triggers a delayed effect on spinogenesis. Additionally, we detected low pMYPT1 levels during the stress and recovery periods as a readout of ROCK activity in synaptoneurosomes. Although there is no study evaluating the presence and function of this regulatory subunit of the phosphatase in spine morphology, it is possible that under this condition, there is a reduction of the phosphorylated forms of non-muscle myosin forms that favor the disruption between acto-myosin interactions and hence, change the protrusive motility of spines (Ryu et al., 2006). The reduction in pMYPT1 levels may increase the activity of MLC phosphatase, thereby favoring acto-myosin relaxation, and probably, allowing spine growth. Interestingly, we have recently reported that after chronic stress, reduction of spine density in CA1 neurons correlates with a rise in pMYPT1, an effect that was prevented by a ROCK inhibitor (Castañeda et al., 2015; Garcia-Rojo et al., 2017). On the other hand, reduced ROCK activity may also reduce the levels of pMLC; an effect that may lead to the formation of filopodia-like spine protrusions from the dendritic shaft (Hodges et al., 2011). Of interest, we found that changes that favored actin filament dynamics preceded the rise in spine density detected 6 h after stress. Furthermore, the changes in spine density were not accompanied by changes in PSD-95, which may indicate that these new spines are labile (Taft and Turrigiano, 2014).

Concomitant with the rise in immature dendritic spine density, after 6 h of recovery from the stress session, the animals showed a DI value near zero, which was significantly different to that of stressed animals. This result indicates that animals did not show preference for novel or familiar object location; i.e., they showed behavior similar to that observed during the training phase. This suggests that the preference of the rat to explore the novel object is beginning to recover 6 h after stress. On the other hand, we observed a significant reduction in the levels of NR1—the constitutive NMDA receptor subunit—in both the homogenate and synaptoneurosome fractions. It is well known that this NMDAR subunit is normally produced in excess and is required for the expression of NMDA receptors at the cell surface (García-Gallo et al., 2001).

The recovery of object location preference occurred with no changes in GluA1, but with an increase in both GluA2 levels and the GluA2/GluA1 ratio in hippocampal synaptoneurosomes; changes that were not observed in the homogenate. This may indicate that the stress recovery period somehow triggers the trafficking of GluA2 receptor subunits to the synapse. One report has indicated that after 1 h of small platform stress, the densities of long-thin and mushroom spines increase, accompanied by colocalization of GluA2 and PSD-95 in spines of CA1 neurons (Sebastian et al., 2013). Moreover, overexpression of GluA2 promotes spinogenesis (Saglietti et al., 2007) and increases spine size and density in hippocampal primary culture neurons (Passafaro et al., 2003). Thus, the stress-induced increase in spine density that we observed could be causally related to the rise in GluA2 expression. On the other hand, facilitated learning by stress is accompanied by enhanced synaptic expression of GluA2 AMPARs that is not observed in mice trained under less stressful conditions (Conboy and Sandi, 2010). Furthermore, GluA2 subunits determine several electrophysiological properties of AMPA receptors, including receptor kinetics, conductance, Ca^2+^ permeability and sensibility to channel blockage by endogenous polyamines (Wright and Vissel, 2012). Therefore, increased GluA2 levels and immature spine morphology may reflect actively-remodeling plastic synapses induced by acute stress exposure. Additionally, changes in GluA2 expression levels may exert these effects by reducing Ca^2+^-permeability of AMPA receptors (Wright and Vissel, 2012), and perhaps, may provide a neuroprotective effect against stress-induced glutamate release (Moghaddam et al., 1994; Popoli et al., 2011).

### Short-Term Memory Recovery Is Accompanied by Restauration of Spine Density and Synaptic Markers

Our experiments show that the impairment in location memory induced by acute stress is gradually recovered; reaching control values 24 h after stress. These results suggest that during the time elapsed between the stress exposure and memory task, several modifications that are able to restore the functionality of the hippocampus may occur. Considering that LTP and LTD are related to spine growth and retraction, respectively (Lai and Ip, 2013), we decided to contrast dendritic spine density in CA1 neurons after 24 h of stress with that observed in control animals. We determined that short-term memory is recovered 24 h after the stress exposure and that spine density, along with the levels of NMDA and AMPA receptor subunits are also recovered to control values. Altogether, these data indicate that acute stress produces reversible molecular and behavioral changes observable after 24 h of stress, allowing short-term hippocampal memory.

## Concluding Remarks and Future Directions

This work is a first effort to determine the multifactorial changes associated with stress exposure and provides evidence that in rats, a single stress exposure differentially produces a transient impairment of hippocampal memory, along with variations in the expression of AMPA and NMDA receptor subunits. These changes are also accompanied by transient changes in spine density and signaling related to actin polymerization. This would indicate that acute stress regulates homeostatic synaptic plasticity mechanisms related to glutamate neurotransmission, which may explain the functional recovery of the hippocampus. Furthermore, our findings open new avenues of research to understand how a single stress exposure may trigger a fast-adaptive response, and how it may be modified during a maladaptive trajectory of stress response, thereby predisposing an individual to neuropsychiatric diseases. Future studies should be conducted to determine the causal relationship between changes in memory, number and morphology of spines and changes in actin dynamics; challenge that requires the availability of specific drugs to intervene the routes or, alternatively, the use of animals with conditional tissue specific *knock*-down. Finally, these results will contribute to the search and discovery of new targets for treating many hippocampal-related disorders derived from maladaptive responses to stress.

## Author Contributions

FIA, MT-B, GD-V, AP, GG-R and JLF designed and performed experiments. FIA, MT-B, GD-V, FAO, WC, LR-A and JLF analyzed and interpreted the data. FIA, JU, EA and JLF wrote the article. JLF, PR and AA critically revised the manuscript. AA conducted the proofreading of the manuscript. All authors edited drafts and approved the final version.

## Conflict of Interest Statement

The authors declare that the research was conducted in the absence of any commercial or financial relationships that could be construed as a potential conflict of interest.
